# Use of a Constrained Acetabular Liner to Prevent and Treat Recurrent Dislocation after Total Hip Replacement Arthroplasty

**DOI:** 10.1111/os.12811

**Published:** 2020-10-25

**Authors:** Joo Hyoun Song, Won Hwan Kwon, Seung‐Bae Oh, Kyoung Ho Moon

**Affiliations:** ^1^ Department of Orthopedic Surgery The Catholic University, St. Vincent's Hospital College of Medicine Suwon‐si South Korea; ^2^ Department of Orthopaedic Surgery Inha University Hospital College of Medicine Incheon South Korea

## Abstract

The aim of the present study was to evaluate the dislocation rate and the risk factors leading to instability after primary and revision total hip replacement arthroplasty (THRA) with constrained acetabular liners (CAL), as well as treatment strategies for prevention of dislocation. From 1999 to 2017, drawing on two institutions' THRA registries, we retrospectively identified 46 THRA cases using a CAL that had been followed up for a minimum of 4 years. The patients comprised 39 women and 7 men, with an average age of 69.1 years (age range, 41–98). Of the 46 patients, CAL were used in 12 patients for prevention of dislocation in primary THRA and in 34 patients for treatment of recurrent dislocation after primary THRA. Clinical and radiological evaluation were performed. We evaluated the failure rate of CAL as well as the risk factors. The 12 patients who used CAL for prevention of dislocation in primary THRA had no dislocation. However, 12 (35%) of the 34 hips had a dislocation after use of CAL in revision THRA. Patients with an abductor muscle weakness grade of ≤3 had a higher rate of dislocation than those with a grade of ≥4 (grade 1; likelihood ratio = ∞, grade 2; likelihood ratio = 1.83, grade 3; likelihood ratio = 1.05, grade 4; likelihood ratio = 0.46, and grade 5; likelihood ratio = 0). The group of primary THRA with CAL had no dislocations, and this is a proper way for prevention of dislocation in high‐risk patients. The group of revision THRA with CAL had a high dislocation rate (35%). Abductor muscle weakness below grade 3 was a risk factor for failure of CAL for hip dislocation. We recommend treating patients with recurrent dislocations with the presence of abductor muscle weakness below grade 3 with not only THRA using CAL but also applying additional abductor muscle reconstruction to reduce the risk of dislocation.

## Graphical abstract

This study was performed to evaluate failure rates and risk factors leading to instability after THRA with constrained acetabular liners (CAL).

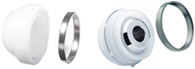

## Introduction

Dislocation is the most common complication after total hip replacement arthroplasty (THRA) and occurs in 1% to 5% of patients after primary THRA. Furthermore, a dislocation incidence of up to 25% after revision THRA has been reported^1^, ^2^, ^3^, ^4^, ^5^. The first step toward resolving instability is identifying the cause. Typical causes include component malposition, abductor muscle deficiency, and neuromuscular disorders^1^.

Several methods are available to correct instability. Nonsurgical methods can be used after a first dislocation through conducting closed reduction and fixing instability temporarily by restricting mobilization using a knee immobilizer, abduction orthoses, or a hip spica cast^6^, ^7^, ^8^. However, revision surgery is recommended in cases of recurrent instability, irreducible dislocation or subluxation, or daily life limitation^9^, ^10^. Various surgical methods have been established, such as correcting malpositioned components, using a liner augmentation wedge or bore components, increasing the head size, using a jumbo head, removing impinging tissue, or reconstructing bone or soft tissue^11^, ^12^, ^13^, ^14^, ^15^, ^16^, ^17^, ^18^, ^19^. In the last 20 years, dual‐mobility bearing and constrained acetabular liners (CAL) have been commonly used^20^. CAL were first introduced to resolve instability in hip joint tuberculosis patients when all previous attempts had failed^21^. The device locks the femur head in acetabular cup components, therefore providing stability^22^.

Although the use of a CAL has been reported to result in treatment successes and dislocation prevention, failure rates are reported between 6% and 42%^23^, ^24^, ^25^. However, few studies have investigated the risk of re‐revision after CAL failure. We hypothesized that the use of CAL in primary THRA and revision THRA would decrease the dislocation rate for patients with high risk of hip dislocation. The aims of the present study were: (i) to investigate the rates of dislocation in the use of CAL for primary THRA and revision THRA and to evaluate the usefulness of THRA using CAL; (ii) to identify risk factors of dislocation in revision THRA with CAL; and (iii) to recognize appropriate treatment strategies following failure to prevent dislocation even using CAL.

## Materials and Methods

### *Inclusion and Exclusion Criteria*

This retrospective study was with the approval of the ethics committee of our institute. Patients who underwent THRA from 1999 to 2017 in two institutions were recruited for the study.

Inclusion criteria were: (i) patients who had undergone primary THRA with CAL with abductor muscle weakness or a neuromuscular disorder (e.g., Parkinson's disease, post‐polio syndrome, cerebral palsy, or residual weakness after stroke); (ii) patients who had undergone revision THRA with CAL who were diagnosed with instability based on ≥3 dislocation episodes attributed to component malposition, abductor muscle weakness, or a neuromuscular disorder. Patients were excluded based on the following criteria: (i) primary THRA without CAL; and (ii) primary THRA and revision THRA patients who were not followed up for at least 4 years (four patients).

### *Patient Information*

**Fig. 1:**
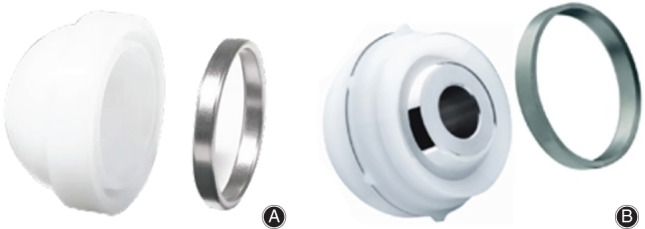
(A) The DURALOC Constrained Liner is indicated for use in total hip replacement arthroplasty cases in which dislocation is a significant postoperative concern. A titanium alloy reinforcing ring strengthens the construct by locking into a circumferential groove in the liner face and securing the prosthetic head through stable axial capture. (B) The PINNACLE Constrained Liner System is designed to address hip instability and provide resistance to dislocation and high ranges of motion with simple, reproducible insertion instruments.

We identified 46 THRA cases using a CAL. Patients were followed up for a minimum of 4 years (a mean follow up of 5.2 years [4–13 years]). The patients comprised 39 women and 7 men, with an average age of 69.1 years (age range, 41–98) (Table 1). In 12 of the 46 patients, a CAL had been used during primary THRA (10: Duraloc [Johnson & Johnson, Warsaw, IN, USA]; 2: Pinnacle [DePuy, Warsaw, IN, USA]) to prevent dislocation, and in the other 34, a CAL had been used to treat recurrent dislocation (29: Duraloc [Johnson & Johnson]; 5: Pinnacle [DePuy, Warsaw]) (Fig. 1A,B). Clinical and radiological evaluations were performed retrospectively.

**TABLE 1:** Patient characteristics

Characteristic	
Age at THRA using constrained liner (year) (range)	69.1 ± 12.1 (41‐98)
Gender	
Female	39 (84.8%)
Male	7 (15.2%)
Height (cm)	155.2 ± 8.1
Weight (kg)	56.6 ± 10.9
BMI (kg/m^2^)	23.4 ± 3.5
BMD (T‐score) (g/cm^2^)	‐3.2 ± 1.2
Number of previous hip surgeries	0.9 ± 1.0
Mean follow up period (month)	62.7 ± 21.0
Reason for hip arthroplasty	
AVN	5 (10.9%)
Hip dysplasia with osteoarthritis	4 (8.7%)
Fracture	37 (80.4%)
Mean number of dislocations (range)	0.7 ± 1.3 (0‐4)

**Fig. 2:**
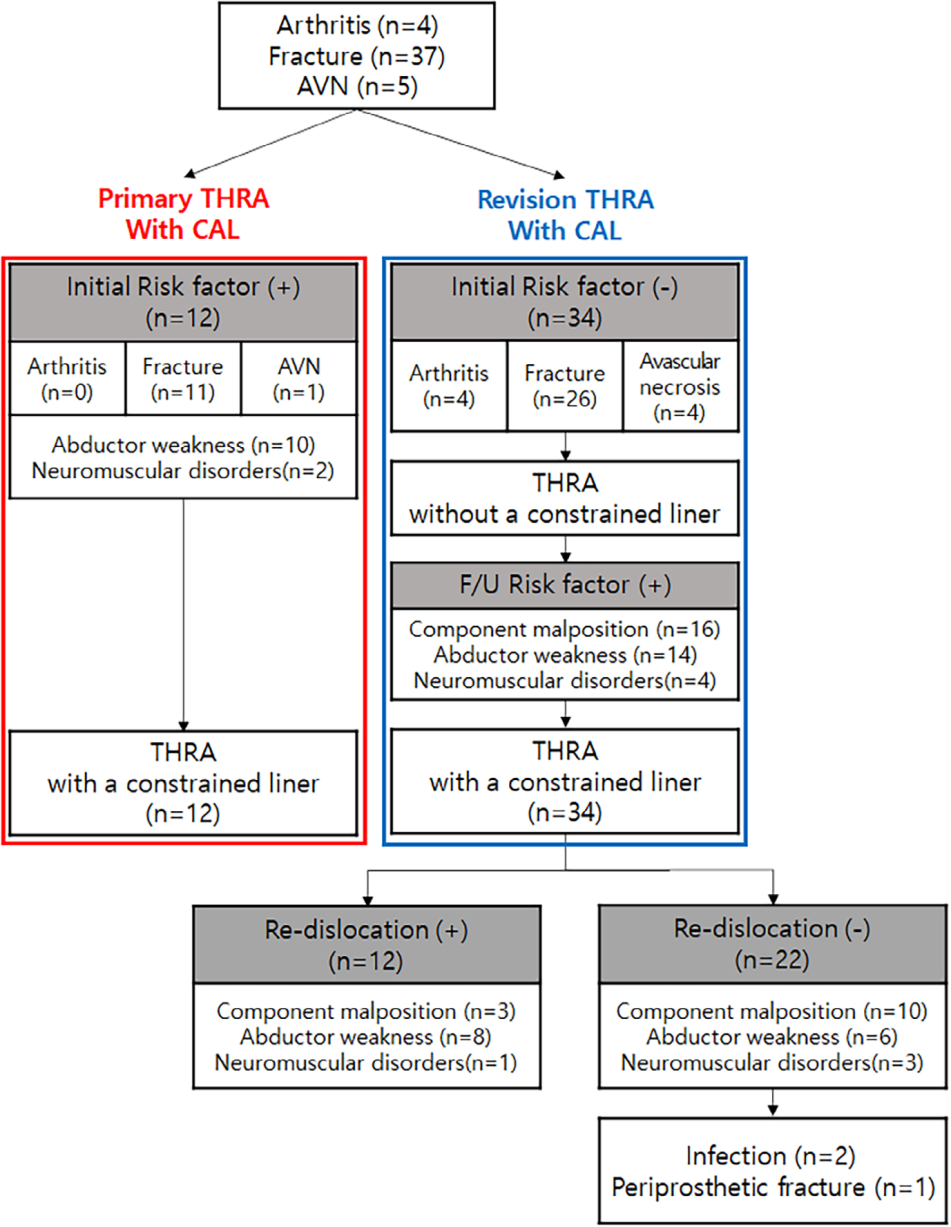
Study flowchart. According to the timing of when constrained acetabular liners (CAL) was used, 12 patients underwent primary total hip replacement arthroplasty (THRA) and the other 34 patients underwent revision THRA for treatment for recurrent dislocation. We divided the 34 revision THRA patients into a successful group (22 patients) and a failed group (12 patients), and then compared and analyzed these two groups.

Preoperative diagnoses of the 46 study subjects were hip arthritis in 4, hip fracture in 37, and avascular necrosis in 5. According to the timing of when CAL was used, 12 patients had undergone primary THRA and the other 34 patients had undergone revision THRA for treatment for recurrent dislocation (Fig. 2).

Among the 34 revision THRA patients, 16 (47%) had component malposition, 14 (41%) had abductor muscle weakness, and 4 (12%) had a neuromuscular disorder. Of the 16 patients with component malposition, 3 had undergone acetabular component with head/liner exchange, and 13 had undergone head/liner to CAL exchange. The patients with abductor muscle weakness or a neuromuscular disorder only underwent head/liner to CAL exchange.

**Fig. 3:**
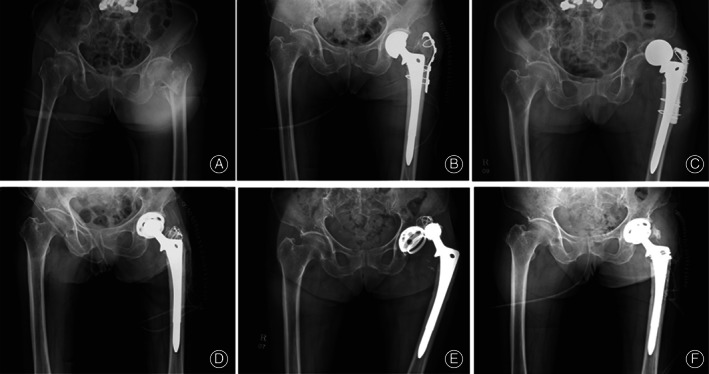
A 71‐year‐old woman who underwent abductor muscle reconstruction using an allo‐Achilles tendon to treat abductor muscle deficiency. (A) Initial preoperative anteroposterior (AP) hip radiograph. (B) Postoperative AP hip radiograph immediately after bipolar hemi arthroplasty (BPHA). (C) AP hip radiographs 3 months after BPHA (dislocation state). (D) Postoperative AP hip radiograph after revision total hip replacement arthroplasty (THRA) using constrained acetabular liners (CAL). (E) AP hip radiographs 2 years after revision THRA (dislocation state). (F) AP hip radiographs after re‐revision THRA with abductor muscle reconstruction using an allo‐Achilles tendon.

Of the 34 revision THRA patients, 12 experienced re‐dislocation after revision surgery. Among these 12 patients, 3 experienced component malposition and underwent acetabular component exchange. Of 8 of the 12 with abductor muscle weakness, 3 underwent a femoral head size exchange from 28 to 36 mm, 1 underwent abductor muscle reconstruction using an allo‐Achilles tendon to treat abductor muscle deficiency (Fig. 3), and 4 underwent a change to a dual‐mobility implant. One patient with a neuromuscular disorder underwent a change to a dual‐mobility implant.

We divided the 34 revision THRA patients into a successful group (22 patients) and a failed group (12 patients), and then compared and analyzed these two groups (Fig. 2). Clinical and radiological evaluations were performed on two groups before and directly after operations, and at 6 weeks, 3 months, 6 months, and 12 months, and annually thereafter.

### *Surgical Technique*

All procedures were performed under general or spinal anesthesia with the patient in the lateral decubitus position. All procedures were performed *via* a posterolateral approach. Incision was started at the posterosuperior border of the greater trochanter and extended proximally for approximately 10 cm toward the posterior superior iliac spine. The fascia lata and ITB were incised longitudinally and proximally to split along the fibers of the gluteus maximus. A Charnley retractor can be placed to hold retraction of the split gluteus maximus. Deep dissection proceeds with hip internal rotation and identification of the piriformis and the other short external rotators (SER). SER are then detached from the greater trochanter close to their insertion. They are reflected posteriorly to both protect the nearby sciatic nerve and expose the posterior hip capsule. After capsulotomy, the surgical site was exposed. A CAL component (Duraloc, Johnson & Johnson/Pinnacle, DePuy) was used in primary THRA or revision THRA. Patients were encouraged to conduct early mobilization and limb exercises after surgery, especially hip abduction function exercises. All patients were allowed to stand within 2 days after removing a hemovac, and they walked with partial weight‐bearing for the first 6 weeks. Then, gradually progressive full weight‐bearing was allowed depending on the stability of the CAL.

### *Outcome Measures*

#### *Evaluation of Harris Hip Scores, Impingement, and Thigh Pain*

Clinical evaluations were performed at final follow ups using Harris hip scores (HHS)^26^. The HHS was developed for the assessment of the results of hip surgery, and is intended to evaluate various hip disabilities and methods of treatment. The domains covered are pain, function, absence of deformity, and range of motion. The score has a maximum of 100 points (best possible outcome), where a score of ≥90 is defined as excellent, ≥80 and <90 as good, ≥70 and <80 as fair, and <70 as poor.

In addition, we assessed whether patients had femoroacetabular impingement at final follow‐ups. Femoroacetabular impingement was defined by Malik *et al* ^27^. Femoroacetabular impingement is the “abutment between the metal femoral neck and the cup liner or between the greater trochanter and pelvis”.

Thigh pain at final follow ups was assessed. We used the definition of Barrack *et al*.^28^, in which thigh pain was present only if a pain drawing showed that the shaded area was on the anterior view and below the inguinal area. The shaded area over the posterior thigh or gluteal region alone was not considered thigh pain, nor was pain that radiated all the way to the toes.

#### *Evaluation of Inclination and Anteversion*

Radiological evaluations were performed using plain hip radiographs (anteroposterior, table‐down view).

Inclination angles were measured as described by Murray *et al* ^29^. The radiological inclination is the “angle between the longitudinal axis and the acetabular axis when projected on to the coronal plane”.

Anteversion angles are described by Murray *et al*.^29^ The radiological anteversion is the “angle between the long axis of the ellipsoid projection of the base of the component and a vertical line”.

Inclination and anteversion angles were classified based on the implant position in the safety zone, as described by Lewinnek *et al* ^30^. Component positions were classified as properly positioned when both inclination and anteversion angles were within the safe zone and as malpositioned when either was outside the safe zone.

#### *Predictors for Recurrent Dislocation*

We conducted statistical analyses of the two groups based on two patient‐related parameters: age, gender, body/mass index (BMI) and initial dislocation time. We also statistically analyzed parameters that may cause instability. We conducted statistical analysis based on whether a patient had component position, neuromuscular disorders, infection, or periprosthetic fractures.

For the analysis of abductor muscle weakness, we subdivided muscle powers by grade (0 to 5), as described by Williams^31^: grade 0, complete paralysis; grade 1, flicker of contraction present; grade 2, active movement with gravity eliminated; grade 3, active movement against gravity; grade 4, active movement against gravity and some resistance described as poor, fair, or moderate strength; and grade 5, normal power.

#### *Dislocation‐Free Survival (All‐Cause/Abductor Muscle Deficiency)*

We evaluated dislocation‐free survival for each cause of instability in all 46 patients and compared dislocation‐free survival with the presence of abductor muscle deficiency.

### *Statistical Analysis*

The statistical analysis was performed using SPSS (Version 19, Chicago, IL, USA) and MedCalc (Version 19.4.0, Ostend, Belgium) software packages for Windows. Cumulative risks of dislocation were estimated using the Kaplan–Meier (KM) method. Associations between patient or procedural characteristics and failure were analyzed using bivariate Cox regression analysis and the χ^2^‐test. Statistical significance was accepted for *P*‐values <0.05.

## Results

### *Outcome Evaluation*

#### *Evaluation of Harris Hip Score, Impingement, and Thigh Pain*

None of the 12 patients that underwent primary THRA experienced dislocation. Of the 34 patients that underwent revision THRA, 22 patients were treated successfully without dislocation (the successful group), and 12 patients (35%, the failed group) experienced treatment failure due to re‐dislocation. These 12 patients and 2 infection and 1 periprosthetic fracture patient in the successful group underwent re‐revision. (Fig. 2).

In the failed group, there were 0 excellent or good, 1 fair (8.3%), and 11 poor (91.7%) HHS. In the successful group, there were 4 excellent (18.2%), 4 good (18.2%), 6 fair (27.3%), and 8 poor (36.4%) HHS. There was no significant correlation between HHS and re‐dislocation incidence (excellent, *P* = 0.424; good, *P* = 0.937; fair, *P* = 0.947; and poor, *P* = 1.000). Femoroacetabular impingement occurred in 2 patients (16.7%) in the failed group and in 1 patient (4.5%) in the successful group. No significant correlation was found between impingement and re‐dislocation incidence (*P* = 0.534). In addition, 4 patients (33.4%) in the failed group and 7 patients (31.8%) in the successful group had thigh pain, but thigh pain was not significantly correlated with re‐dislocation (*P* = 0.662) (Table 2).

**TABLE 2:** Evaluation of HHS, impingement and thigh pain

Factor	Failed group (N=12)	Successful group (N=22)	*P* value
Harris hip score			
Excellent	0 (0%)	4 (18.2%)	0.424
Good	0 (0%)	4 (18.2%)	0.937
Fair	1 (8.3%)	6 (27.3%)	0.947
Poor	11 (91.7%)	8 (36.4%)	1.000
Impingement	2 (16.7%)	1 (4.5%)	0.534
Thigh pain	4 (33.4%)	7 (31.8%)	0.662

#### *Evaluation of Inclination and Anteversion*

The failed group had 1 case (8.3%) with an anteversion angle <5°, 10 cases (83.3%) with an angle between 5° and 25°, and 1 case (8.3%) with an angle >25°. The successful group had 5 cases (22.7%) with an anteversion angle <5°, 12 cases (54.5%) with an angle between 5° and 25°, and 5 cases (22.7%) with an angle >25°. No significant correlation was found between the anteversion angle and re‐dislocation for angles <5° (hazard ratio = 0.42; *P* = 0.407) or >25° (hazard ratio = 0.28; *P* = 0.227). The failed group had 1 case (8.3%) with an inclination angle under 30°, 11 cases (91.7%) with an angle between 30° and 50°, and 0 cases (0%) with an angle of >50°. The successful group had 0 cases (0%) with an inclination angle <30°, 19 cases (86.4%) with an angle between 30° and 50°, and 3 cases (13.6%) with an angle >50°. The inclination angle was not significantly correlated with re‐dislocation whether <30° (hazard ratio = 2.5; *P* = 0.388) or >50° (hazard ratio = 0.0; *P* = 0.985) (Table 3).

**TABLE 3:** Evaluation of inclination and anteversion

Factor	Failed group (N = 12)	Successful group (N = 22)	Hazard Ratio	*P* value
Inclination				0.689
<30º	1 (8.3%)	0 (0%)	2.5	0.388
30º‐50º	11 (91.7%)	19 (86.4%)	Reference	
>50º	0 (0%)	3 (13.6%)	0.0	0.985
Anteversion				0.376
<5º	1 (8.3%)	5 (22.7%)	0.42	0.407
5º‐25º	10 (83.3%)	12 (54.5%)	Reference	
>25º	1 (8.3%)	5 (22.7%)	0.28	0.227

#### *Predictors for Recurrent Dislocation*

##### Patient‐Related Parameters

In the failed group, 11 (91.6%) patients were <75 years old, whereas in the successful group, 14 (63.6%) were <75 years old. No significant correlation was observed between age (<75 years old) and re‐dislocation (hazard ratio = 4.5, 95% confidence interval; *P* = 0.146). In the failed group, 10 (83.3%) patients were female, whereas in the successful group, 19 (86.4%) were female. No significant correlation was observed between gender and re‐dislocation (hazard ratio = 0.79, 95% confidence interval; P = 0.406). In the failed group, 5 patients (91.6%) had a BMI <25 m^2^/kg and 1 (8.3%) had a BMI of >30. In the successful group, 14 (63.6%) had a BMI of <25 and 1 (4.5%) had a BMI of >30. No significant correlation was observed between BMI and re‐dislocation for patients with BMI <25 (hazard ratio = 0.46; *P* = 0.194) or >30 (hazard ratio = 0.99; *P* = 0.989). In the failed group, 10 (83.3%) patients initially experienced dislocation at 1 year or less, whereas in the successful group, 16 (72.7%) initially experienced dislocation at 1 year or less. No significant correlation was observed between initial dislocation time and re‐dislocation (hazard ratio = 1.88, 95% confidence interval; P = 0.245). (Table 4).

**TABLE 4:** Patient‐related risk factors of re‐dislocation

Factor	Failed group (N = 12)	Successful group (N = 22)	Hazard Ratio	*P* value
Age				
75 year or more	1 (8.3%)	8 (36.4%)	Reference	
<75 year	11 (91.6%)	14 (63.6%)	4.57	0.146
Gender				
Male	2 (16.7%)	3 (13.6%)	Reference	
Female	10 (83.3%)	19 (86.4%)	0.79	0.406
BMI				0.164
<25 kg/m^2^	5 (41.7%)	14 (63.6%)	0.46	0.194
25 kg/m^2^ ‐ 30 kg/m^2^	6 (50%)	7 (31.8%)	Reference	
>30 kg/m^2^	1 (8.3%)	1 (4.5%)	0.99	0.989
Initial dislocation time				
>1 year	2 (16.7%)	6 (27.3%)	Reference	
1 year or less	10 (83.3%)	16 (72.7%)	1.88	0.245

##### Instability Parameter

In the failed group, 3 (25%) had component malposition, whereas in the successful group, 10 (45.5%) had component malposition. No significant correlation was observed between component malposition and re‐dislocation (hazard ratio = 0.49; *P* = 0.295). In the failed group, 1 (8.3%) had a neuromuscular disorder, and in the successful group, 3 (13.6%) had a neuromuscular disorder. No significant correlation was found between neuromuscular disorder and re‐dislocation (hazard ratio = 0.557; *P* = 0.575). In the successful group, infection or fracture occurred in 2 (9.1%) and 1 (4.5%) case, respectively. Neither variable was significantly correlated with re‐dislocation (infection: hazard ratio = 0.044; *P* = 0.534 and periprosthetic fracture: hazard ratio 0.047; *P* = 0.662) (Table 5).

**TABLE 5:** Instability‐related risk factors of re‐dislocation

Factor	Failed group (N=12)	Successful group (N=22)	Hazard ratio	*P* value
Component malposition	3 (25%)	10 (45.5%)	0.49	0.295
Neuromuscular disorder	1 (8.3%)	3 (13.6%)	0.557	0.575
Infection	0	2 (9.1%)	0.044	0.534
Periprosthetic fracture	0	1 (4.5%)	0.047	0.662

For abductor muscle weakness, in the failed group, there were 2 cases (16.7%) of grade 0, 2 (16.7%) of grade 1, 2 (16.7%) of grade 2, 4 (33.4%) of grade 3, 2 (16.7%) of grade 4, and no cases of grade 5. In the successful group, there were 0 cases (0%) of grade 0, 0 cases (0%) of grade 1, 2 cases (9.1%) of grade 2, 7 cases (31.8%) of grade 3, 8 cases (36.4%) of grade 4, and 5 cases (22.7%) of grade 5. Notably, abductor muscle weakness was significantly correlated with re‐dislocation. Patients with an abductor muscle weakness grade of ≤3 had a higher rate of dislocation than those with a grade of ≥4 (grade 1, likelihood ratio = ∞; grade 2, likelihood ratio = 1.83; grade 3; likelihood ratio = 1.05; grade 4, likelihood ratio = 0.46; and grade 5, likelihood ratio = 0) (Table 6).

**TABLE 6:** Abductor muscle weakness‐related risk factors of re‐dislocation

Factor	Failed group (N=12)	Successful group (N=22)	Likelihood Ratio	95% CI
Abductor weakness				
Grade 0	2 (16.7%)	0 (0%)	∞	0.36 to ∞
Grade 1	2 (16.7%)	0 (0%)	∞	0.36 to ∞
Grade 2	2 (16.7%)	2 (9.1%)	1.83	0.29 to 11.42
Grade 3	4 (33.4%)	7 (31.8%)	1.05	0.38 to 2.87
Grade 4	2 (16.7%)	8 (36.4%)	0.46	0.12 to 1.82
Grade 5	0 (0%)	5 (22.7%)	0	0.00 to 3.08

#### *Dislocation‐Free Survival (All‐Cause/Abductor Muscle Deficiency)*

**Fig. 4:**
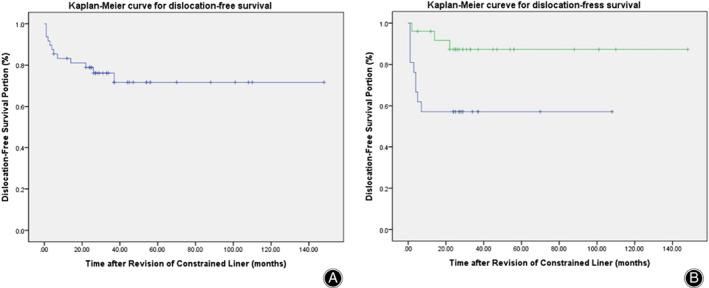
(A) Kaplan–Meier curve showing dislocation‐free survival rates for all causes. Twelve‐month and final follow‐up dislocation‐free survival rates were 78.2% and 73.6%, respectively. (B) Kaplan–Meier curves showing dislocation‐free survival rates based on whether patients had abductor muscle deficiency. (Blue: abductor muscle deficiency. Green: no abductor muscle deficiency.) In cases with abductor muscle deficiency, dislocation‐free survival rates were 61.9% at 6 months and 57.1% at 12 months, and in cases without abductor muscle deficiency, dislocation‐free survival rates were 96.0% at 6 months, 94.7% at 12 months, and 87.3% at final follow‐up.

For all 46 study subjects, dislocation recurred in 12 cases, and KM‐estimated cumulative dislocation‐free survival rates were 78.2% at 12 months and 73.6% at 24 months (Fig. 4A). In addition, we compared KM‐estimated cumulative dislocation‐free survival rates according to the presence of abductor muscle deficiency. In cases of abductor muscle deficiency, dislocation‐free survival rates were 61.9% at 6 months and 57.1% at 12 months, whereas in cases of no abductor muscle deficiency, dislocation‐free survival rates were 96.0% at 6 months, 94.7% at 12 months, and 87.3% at final follow up. These results confirmed that patients with abductor muscle deficiency had a higher rate of re‐dislocation (Fig. 4B).

### *Complications*

Among the 34 patients, 2 underwent re‐revision due to infection and were treated by irrigation and debridement followed by acetabular and femoral component changes with two‐stage surgery. One patient with a periprosthetic fracture, diagnosed as a Vancouver type B3 periprosthetic femoral fracture, which occurred 46 months after the original CAL procedure, underwent a femoral component change and open reduction and internal fixation using a plate and wire.

## Discussion

### *Evaluation of Usefulness of Constrained Acetabular Liners*

Few studies have reported dislocation results for high‐risk patients who have undergone primary THRA using a CAL to prevent dislocation. In this study, no dislocation occurred in patients who had undergone primary THRA using a CAL, which shows that use of a CAL is a good option for preventing dislocation in high‐risk patients.

In previous studies, the use of a CAL to correct instability was associated with relatively high rates of re‐dislocation. Daly and Morre *et al*. reported a re‐dislocation rate of 39% in 95 cases after a 7.6‐year follow up^11^. Carter *et al*. reported re‐dislocation in 21% of 156 cases after 67 months^32^, and Wetter *et al*. reported a failure rate of 19% for 129 cases^33^. In the present study, we observed 12 cases of re‐dislocation among 34 revision THRA cases using a CAL, which is similar or somewhat higher than rates previously reported. We suggest that these differences were probably due to different implant types, surgical methods, and/or patient differences.

We found no significant difference in the HHS between the primary THRA group and the revision THRA group after a minimum follow‐up of 4 years: the average HHS increased by two in the primary THRA group and by three in the revision THRA group. Furthermore, we found no significant difference between the successful group and the failed group. This result is consistent with previous studies in which less improvement was reported in patients that received only modular liner revision than in patients who also received acetabular component revision^34^. Therefore, improvement in clinical symptoms is not expected, although dislocation incidence is reduced by conducting a CAL linear change.

The results of the radiologic evaluations conducted in the present study indicated that acetabular components were located in the safe zone in most cases and that the proportion of acetabular components in the safe zone was higher in the failed group. Recent studies have also revealed that most dislocation incidences occur in Lewinnek's safe zone, and have speculated that this discrepancy results from individual anatomical differences, such as femoral component version or offset, or the position of the acetabular component in perioperative and postoperative postures^35^, ^36^, ^37^. Further research is needed on the dynamic movement position of the acetabular component during postoperative routine daily activities.

### *Risk Factors of Re‐Dislocation*

Previous research has revealed that component malposition, abductor muscle weakness, and neuromuscular disorders are predictors of re‐dislocation after revision THRA. In the present study, we found no correlation between re‐dislocation and patient‐related parameters (age, gender, BMI and initial dislocation time), but analysis of instability parameters showed that abductor muscle weakness was more associated with re‐dislocation than component malposition or the presence of a neuromuscular disorders. We analyzed abductor muscle weakness based on muscle power grade and found a higher rate of hip dislocation in cases with abductor muscle weakness of ≤ grade 3.

### *Treatment Strategies*

In most cases, the abductor muscle is damaged during revision THRA procedures but can be corrected using methods such as muscle repair, grafting, and/or trochanter advancement^38^, ^39^. We recommend treating recurrent dislocation in cases of abductor muscle weakness of ≤ grade 3 by THRA using CAL with additional abductor muscle reconstruction to reduce dislocation risk.

### *Limitations*

The present study has a number of limitations that warrant consideration. First, it was conducted retrospectively with no control group and its retrospective design introduced the possibility of selection bias. Second, it was difficult to objectively verify indications of CAL as assessments and analyses were made using operator records. Third, the number of study subjects was relatively small, and, thus, results may lack statistical significance. We suggest a larger‐scale prospective study be conducted to confirm our findings.

### *Conclusion*

Based on the results of this study we recommend that CAL be used during primary THRA in patients at high risk of dislocation, and that in cases of recurrent dislocation with abductor muscle weakness (especially below grade 3) CAL implantation and additional abductor muscle reconstruction be conducted to reduce the failure rate.
